# Predictive value of hematological parameters for IVF success: a retrospective analysis

**DOI:** 10.3389/frph.2025.1746987

**Published:** 2025-12-18

**Authors:** Fatma Tulucu Kalkan, Ufuk Atlihan, Gökçe Aykanat, Begum Ertan, Ferruh Acet, Ege Nazan Tavmergen Goker, Erol Tavmergen

**Affiliations:** 1 TC Saglik Bakanligi Gaziantep Sehir Hastanesi, Şahinbey, Türkiye; 2 Manisa Merkezefendi State Hospital, Manisa, Türkiye; 3 Kınık State Hospital, Izmir, Türkiye; 4 Akhisar State Hospital, Manisa, Türkiye; 5 Tavmergen IVF Clinic, Izmir, Türkiye; 6 Ege Universitesi Tip Fakultesi, Izmir, Türkiye

**Keywords:** embryo quality, hematologic indices, inflammation, IVF, neutrophil-to-lymphocyte ratio, systemic immune-inflammation index, unexplained infertility

## Abstract

**Objective:**

To evaluate the predictive value of hematologic inflammatory indices derived from complete blood count (CBC) parameters for *in vitro* fertilization (IVF) outcomes in women with unexplained infertility, and to determine their potential role as biomarkers of subclinical inflammation affecting reproductive success.

**Materials and methods:**

This retrospective cohort study included 430 women with unexplained infertility who underwent IVF/ICSI cycles at Ege University Hospital between January 2020 and January 2025. CBC parameters and derived systemic inflammatory indices—including neutrophil-to-lymphocyte ratio (NLR), platelet-to-lymphocyte ratio (PLR), monocyte-to-lymphocyte ratio (MLR), systemic immune-inflammation index (SII), systemic inflammation response index (SIRI), and pan-immune-inflammation value (PIV)—were measured on the ovulation trigger day. Patients were divided into pregnant and non-pregnant groups according to clinical pregnancy outcomes. Demographic, hormonal, hematologic, and embryologic variables were compared using appropriate statistical tests, and logistic regression and ROC analyses were performed to identify independent predictors of pregnancy.

**Results:**

None of the CBC-derived inflammatory indices showed significant differences between pregnant and non-pregnant groups (all *p* > 0.05). ROC analysis revealed poor discriminative ability for predicting pregnancy (AUC values 0.48–0.52). In contrast, embryologic variables—particularly the number of two-pronuclei (2PN) embryos and total embryos—were independent predictors of clinical pregnancy (*p* < 0.05), while excessive oocyte yield was inversely associated with pregnancy (*p* = 0.028).

**Conclusion:**

Systemic inflammatory indices derived from CBC parameters, including NLR, PLR, MLR, SII, SIRI, and PIV, do not predict IVF/ICSI outcomes in women with unexplained infertility. Embryologic parameters, especially 2PN and total embryo counts, remain the most reliable predictors of clinical pregnancy.

## Introduction

Infertility, defined as the inability to conceive after 12 months of unprotected intercourse, affects approximately 18% of couples worldwide (1). Assisted reproductive technologies (ART) are widely used in the management of infertility, particularly in cases where multiple contributing factors, including genetic predispositions, are involved (2). Among them, up to 30% are diagnosed with unexplained infertility (UI), a condition characterized by the absence of any identifiable abnormalities in standard fertility evaluations, including ovulation assessment, tubal patency, and semen analysis. In such cases, subtle defects in oocyte quality, tubal transport, or sperm function may exist but remain undetected by conventional diagnostic tools. When conventional treatments fail, *in vitro* fertilization (IVF) is considered a valuable option for couples with UI. Although continuous progress in IVF techniques, the overall pregnancy success rate remains limited, with only about 32% of women achieving pregnancy following the procedure (3). IVF not only facilitates conception but also provides additional insight into gamete competence and early embryo development. Despite its advantages, IVF outcomes in UI patients tend to be less favorable compared to other infertility diagnoses, suggesting the involvement of unrecognized biological or immunological factors that may interfere with reproductive success (4). During the early development of the preimplantation embryo, maternal immune and inflammatory status, cytokine balance, and chronic stress are as influential as genetic factors. A wide range of health conditions and diseases can modify reproductive cytokine activity, either by directly affecting the physiological functions of reproductive tissues or by increasing circulating proinflammatory mediators at the systemic level. These changes indicate that cytokines play a role not only in fertility and the progression of pregnancy but also in determining embryonic health and developmental pathways. Therefore, a comprehensive understanding of cytokine networks regulating the embryo is essential to optimize conception outcomes in both natural and assisted reproductive settings (5). Autoimmunity arises when the immune system fails to properly recognize self from non-self, triggering inflammatory pathways that involve cellular mechanisms, antibody activity, and immune complex formation within both the innate and adaptive immune responses.^1^ Pathogen-associated molecules stimulate the innate immune system, leading to the secretion of inflammatory mediators that subsequently trigger activation of monocytes, macrophages, neutrophils, natural killer cells, and mast cells (6). Alongside leukocytes, platelets are significant contributors to inflammation through their release of mediators that induce site-specific modifications in the inflammatory response (7). Recently, hematologic indices derived from complete blood count (CBC) parameters have been proposed as surrogate markers of systemic inflammation. Among these, the neutrophil-to-lymphocyte ratio (NLR), platelet-to-lymphocyte ratio (PLR), and monocyte-to-lymphocyte ratio (MLR) have shown prognostic value in various clinical settings (8–10). Furthermore, novel composite indices such as the systemic immune-inflammation index (SII), systemic inflammation response index (SIRI), and pan-immune-inflammation value (PIV) have gained attention for their ability to capture the overall immune-inflammatory status in a cost-effective and noninvasive manner (11–13). However, data on the role of these markers in unexplained infertility remains scarce. Whether systemic immune activation contributes to impaired oocyte competence, embryo development, or IVF failure in UI patients is still unclear. This study aims to evaluate the predictive value of CBC-derived inflammatory indices on IVF outcomes in women with unexplained infertility, and to explore their potential role as biomarkers for subclinical inflammation affecting reproductive success.

## Materials and methods

**Figure 1:**
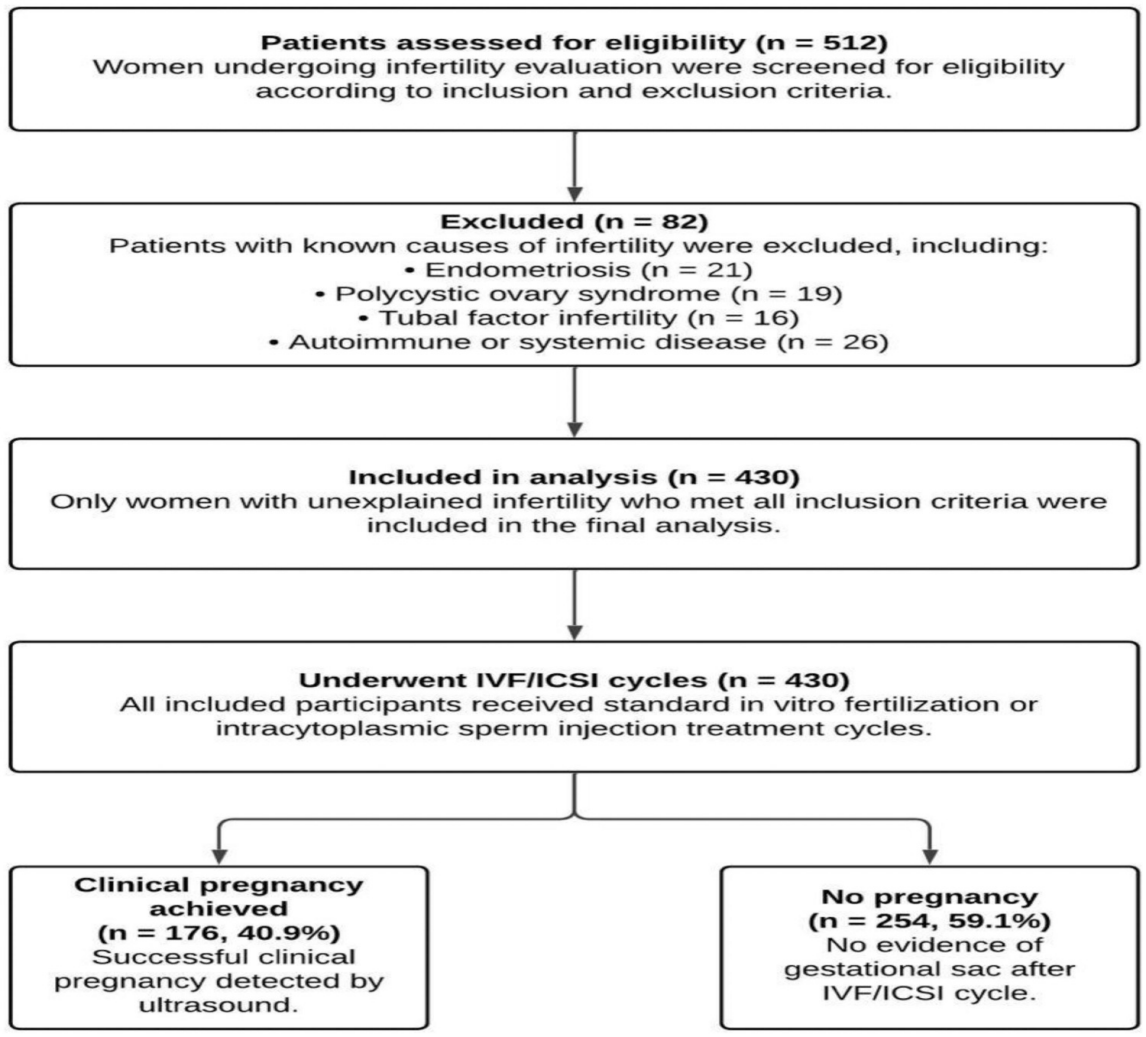
Study flow diagram of patient inclusion and outcomes.

This retrospective cohort study was conducted at the IVF Unit of Ege University Hospital (İzmir, Türkiye) between January 2020 and January 2025. This study was approved by the Ege University Clinical Research Ethics Committee (approval number: 25–6.1T/17, date: 26 June 2025) and conducted in accordance with the Declaration of Helsinki. Given the retrospective design, the requirement for written informed consent was waived by the Ethics Committee. All patient data were de-identified and analyzed anonymously to ensure confidentiality (14). The medical records of women diagnosed with unexplained infertility who underwent IVF/ICSI cycles were reviewed. Patients were divided into two groups according to clinical pregnancy outcome (pregnant vs. non-pregnant) (Figure 1). Unexplained infertility was diagnosed based on the following criteria: regular ovulatory cycles, bilaterally patent fallopian tubes, normal semen parameters, and absence of uterine pathology. For patients who had undergone repeated treatment cycles, only data from the first cycle were analyzed. Women were eligible for inclusion if they met the following criteria: (i) age between 20 and 42 years, (ii) a confirmed diagnosis of unexplained infertility after comprehensive evaluation, and (iii) the availability of complete demographic, hormonal, hematologic, and cycle outcome data.

Exclusion criteria comprised the presence of any systemic diseases that could influence systemic inflammation (including hematologic, endocrine, rheumatologic, or autoimmune disorders), acute infections, or chronic use of glucocorticoids or anti-inflammatory medications. Women with endometriosis, polycystic ovary syndrome (PCOS), hydrosalpinx, acute or chronic infections, autoimmune or rheumatologic diseases, metabolic disorders, chronic glucocorticoid or NSAID use, and cycles cancelled before oocyte retrieval were excluded to avoid confounding inflammatory effects. Patients with tubal factor infertility, male factor infertility, diminished ovarian reserve, or incomplete medical records were also excluded from the study. All patients were managed according to the standard IVF protocol of the Ege University IVF Center. A baseline transvaginal ultrasound was performed on cycle days 2–3 to rule out ovarian cysts or uterine abnormalities. Controlled ovarian stimulation was initiated using gonadotropins (recombinant FSH or highly purified hMG) in combination with a GnRH antagonist protocol. The starting dose and duration of gonadotropins were individualized based on age, antral follicle count, and ovarian reserve. When at least two follicles reached ≥18 mm, final oocyte maturation was triggered with 10,000 IU hCG or 250 µg recombinant choriogonadotropin alfa. Oocyte retrieval was performed 36 h after triggering under ultrasound guidance and conscious sedation. All retrieved oocytes underwent ICSI, and fertilization was confirmed by the presence of two pronuclei (2PN) approximately 16–18 h later. Embryos were cultured to day 3 or day 5 based on morphology and cleavage dynamics. Embryo transfer was performed under transabdominal ultrasound guidance using a soft catheter. Luteal phase support consisted of vaginal micronized progesterone (600–800 mg/day) and oral estradiol valerate (4–6 mg/day), starting on the day of oocyte retrieval and continuing until the pregnancy test. Serum β-hCG levels were measured 12–14 days after transfer, and hormone supplementation was maintained up to 12 weeks of gestation in confirmed pregnancies (β-hCG > 50 mIU/mL). For each patient included in the study, demographic and baseline clinical data were collected, including age, body mass index (BMI), infertility duration, infertility type, and cycle characteristics. Basal hormonal parameters—follicle-stimulating hormone (FSH), luteinizing hormone (LH), estradiol (E2), and anti-Müllerian hormone (AMH)—were recorded on cycle day 3. A complete blood count (CBC) was obtained on the ovulation trigger day, and the following parameters were evaluated: white blood cell (WBC), neutrophil, lymphocyte, monocyte, eosinophil, basophil, platelet, erythrocyte, and hemoglobin levels. From these measurements, the following derived inflammatory indices were calculated: neutrophil-to-lymphocyte ratio (NLR), platelet-to-lymphocyte ratio (PLR), monocyte-to-lymphocyte ratio (MLR), systemic immune-inflammation index (SII = platelet × neutrophil/lymphocyte), systemic inflammation response index (SIRI = neutrophil × monocyte/Lymphocyte), and pan-immune-inflammation value (PIV = neutrophil × platelet × monocyte/lymphocyte). All CBC samples were collected in the morning (07:00–09:00) after overnight fasting to minimize diurnal and nutritional variability. A single standardized venous sample was obtained for all participants. All indices were calculated using validated formulas previously described in the literatüre. Embryologic parameters including total oocyte number, number of mature (MII) oocytes, number of two-pronuclear (2PN) embryos, total embryos were retrieved from the laboratory database. Serum *β*-hCG levels were measured 12–14 days after embryo transfer to determine biochemical pregnancy. Clinical pregnancy was defined as the presence of an intrauterine gestational sac with fetal heartbeat detected by transvaginal ultrasound, and viable pregnancy as persistence of cardiac activity beyond 8 weeks of gestation. The primary outcome of the study was clinical pregnancy, while secondary outcomes included biochemical pregnancy, viable pregnancy, and the association of hematologic and embryologic parameters with pregnancy success. The principal objective of the analysis was to assess the predictive value of CBC-derived systemic inflammatory indices for IVF/ICSI outcomes in women with unexplained infertility, and to identify independent demographic, hormonal, and embryologic predictors of reproductive success.

### Statistical analysis

All statistical analyses were performed using IBM SPSS Statistics for Windows, Version 25.0 (IBM Corp., Armonk, NY, USA). Continuous variables were presented as mean ± standard deviation (SD), while categorical variables were expressed as numbers and percentages (%). The Kolmogorov–Smirnov test was applied to assess the normality of continuous data.

For normally distributed variables, comparisons between groups were conducted using the independent-samples *t*-test, and for non-normally distributed data, the Mann–Whitney *U*-test was employed. Categorical variables were compared using the Chi-square test or Fisher's exact test, as appropriate. A two-tailed *p* value < 0.05 was considered statistically significant. To evaluate predictors of pregnancy, univariate binary logistic regression analyses were initially performed for all candidate variables, including age, BMI, infertility duration, FSH, LH, E2, AMH, total oocyte count, 2PN embryo number, total embryo number, and CBC-derived inflammatory indices (WBC, NLR, PLR, MLR, SII, SIRI, PIV). Variables with *p* < 0.10 in univariate analysis were subsequently included in a multivariate logistic regression model to identify independent predictors of clinical pregnancy. The results were reported as β coefficients, odds ratios (OR), and 95% confidence intervals (CI). In addition, receiver operating characteristic (ROC) curve analyses were conducted to assess the discriminative ability of embryologic variables and inflammatory indices for predicting pregnancy outcomes. The area under the curve (AUC) with 95% CI was calculated for each variable, and the optimal cut-off point was determined using Youden's index (sensitivity + specificity−1). The corresponding sensitivity and specificity values were also reported (15). All analyses were performed under the supervision of a professional biostatistician. Potential confounders were selected *a priori* based on biological plausibility and previous literature. The following variables were included as covariates in the multivariable model: age, BMI, infertility duration, basal FSH, AFC, infertility etiology, and stimulation protocol.

## Results

The mean age of the participants was 32.3 ± 4.3 years and the mean body mass index (BMI) was 25.1 ± 4.5 kg/m^2^. The mean infertility duration was 4.2 ± 2.6 years. The gravidity of the pregnant group was significantly higher compared with the non-pregnant group (*p* = 0.049) (Table 1).

**Table 1:** Demographic and baseline characteristics of the study population.

Variables	Pregnancy (+) (*n* = 176)	Pregnancy (−) (*n* = 254)	*p*-value
	Mea*n* ± SD		
Age (years)	32.1 ± 4.2	32.4 ± 4.4	0.94
BMI (kg/m^2^)	25.0 ± 4.6	25.2 ± 4.4	0.72
Gravidity	1.2 ± 0.8	1.0 ± 0.6	0.049
Parity	0.8 ± 0.4	0.7 ± 0.3	0.317
Infertility duration (years)	4.1 ± 2.5	4.3 ± 2.7	0.41
Smoking (%)	11.4%	12.8%	0.68

*BMI, body mass index.

Baseline hormonal profiles, including FSH (*p* = 0.517), LH (*p* = 0.986), E2 (*p* = 0.398), and AMH (*p* = 0.172), showed no statistically significant differences between pregnant and non-pregnant women. Peripheral complete blood count (CBC) indices measured on the ovulation trigger day—including WBC, neutrophil, lymphocyte, monocyte, eosinophil, basophil, platelet, erythrocyte, and hemoglobin levels—were comparable between groups (all *p* > 0.05).

To further evaluate subclinical inflammation, several derived indices were calculated: neutrophil-to-lymphocyte ratio (NLR), platelet-to-lymphocyte ratio (PLR), monocyte-to-lymphocyte ratio (MLR), systemic immune-inflammation index (SII), systemic inflammation response index (SIRI), and pan-immune-inflammation value (PIV). All indices demonstrated non-significant intergroup differences (Table 2).

**Table 2:** Hematologic and inflammatory indices measured on the ovulation trigger day.

Variables	Pregnancy (+) (n = 176)	Pregnancy (−) (*n* = 254)	*p*-value
	Mean ± SD		
WBC (×10^9^ /L)	7.42 ± 1.88	7.61 ± 1.97	0.106
Neutrophil (×10^9^ /L)	4.39 ± 1.29	4.47 ± 1.36	0.284
Lymphocyte (×10^9^ /L)	2.21 ± 0.64	2.18 ± 0.59	0.413
Monocyte (×10^9^ /L)	0.46 ± 0.12	0.45 ± 0.13	0.478
Eosinophil (×10^9^ /L)	0.19 ± 0.11	0.21 ± 0.13	0.356
Basophil (×10^9^ /L)	0.05 ± 0.02	0.05 ± 0.02	0.517
Platelet (×10^9^ /L)	257.4 ± 61.5	261.8 ± 58.9	0.373
Erythrocyte (×10^12^ /L)	4.49 ± 0.37	4.47 ± 0.35	0.529
Hemoglobin (g/dL)	12.92 ± 0.91	12.86 ± 0.89	0.671
NLR	2.07 ± 0.83	2.12 ± 0.88	0.562
PLR	122.6 ± 46.9	124.3 ± 49.5	0.607
MLR	0.21 ± 0.07	0.20 ± 0.08	0.489
SII	907.3 ± 314.8	924.6 ± 331.2	0.418
SIRI	0.47 ± 0.16	0.49 ± 0.17	0.364
PIV	148.2 ± 59.6	152.8 ± 61.4	0.329

Receiver operating characteristic (ROC) analysis revealed poor discriminative performance for predicting clinical pregnancy: AUC values ranged between 0.48 and 0.52, with the highest observed for SII (AUC = 0.514). Optimal cut-off points determined by the Youden index produced low sensitivity and specificity, and thus lacked clinical utility.

A multivariable logistic regression model incorporating all indices confirmed that none independently predicted pregnancy outcomes (model AUC ≈ 0.57). These findings collectively indicate that systemic inflammatory indices derived from baseline CBC measurements have limited prognostic relevance for IVF success in unexplained infertility.

Embryologic parameters were significantly associated with pregnancy outcomes.

The mean total oocyte count was higher in the pregnant group but only at a borderline significance (*p* = 0.0526). In contrast, the mean numbers of two-pronuclei (2PN) embryos and total embryos were markedly higher among pregnant women (both *p* < 0.001) (Table 3).

**Table 3:** Embryologic outcomes in pregnant and non-pregnant groups.

Variables	Pregnancy (+) (*n* = 176)	Pregnancy (−) (*n* = 254)	*p*-value
	Mean ± SD		
Total oocytes retrieved	10.8 ± 4.3	9.7 ± 4.1	0.0526
MII oocytes	8.9 ± 3.8	8.2 ± 3.7	0.113
2PN embryos	6.1 ± 2.9	4.7 ± 2.6	<0.001
Total embryos	5.3 ± 2.7	3.9 ± 2.4	<0.001
Blastocyst formation rate (%)	41.5 ± 14.2	39.8 ± 13.6	0.273
Embryo quality score	7.8 ± 1.6	7.4 ± 1.7	0.119

**Figure 2:**
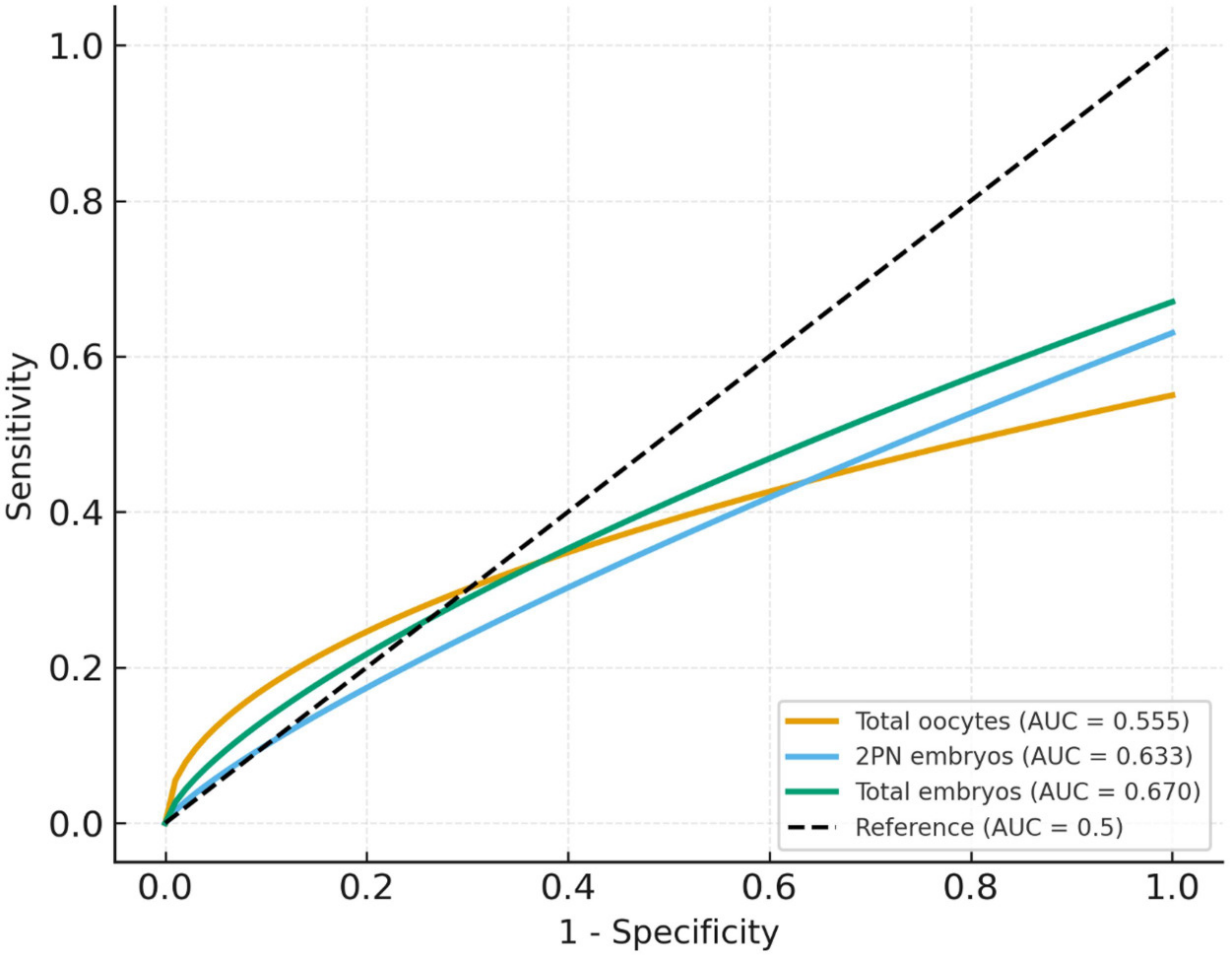
ROC curve analysis of embryological variables for clinical pregnancy.

ROC curve analysis was performed to determine the predictive performance of embryologic variables for clinical pregnancy. Total embryo number demonstrated the highest discriminative power (AUC = 0.670, optimal cut-off ≥2, sensitivity = 0.858, specificity = 0.421). 2PN embryo count also showed moderate discriminative ability (AUC = 0.633, cut-off ≥3, sensitivity = 0.835, specificity = 0.350). Total oocyte count displayed minimal predictive capacity (AUC = 0.555) (Figure 2). These findings confirm that embryo-related variables outperform systemic inflammatory indices in predicting clinical pregnancy, highlighting their superior prognostic relevance.

Collectively, these results suggest that while embryo-related variables hold potential for clinical prediction, their sensitivity remains higher than specificity, indicating they may be better suited for identifying likely successes rather than excluding failures (Table 4).

**Table 4:** ROC analysis of embryologic parameters for prediction of clinical pregnancy.

Predictor	AUC (95% CI)	Optimal cut-off	Sensitivity (%)	Specificity (%)	*p*-value
Total oocytes	0.555 (0.48–0.63)	≥8	67.3	43.9	0.083
2PN embryos	0.633 (0.57–0.70)	≥3	83.5	35.0	<0.001
Total embryos	0.670 (0.61–0.73)	≥2	85.8	42.1	<0.001

**Figure 3:**
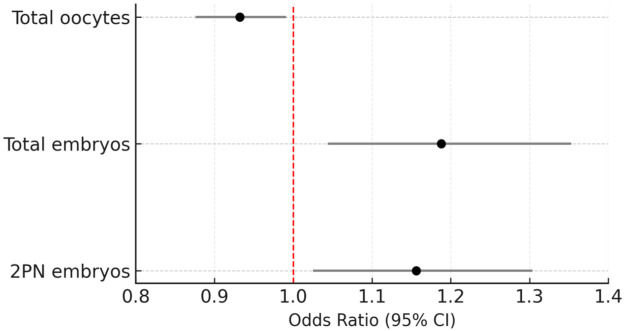
Logistic regression analysis of embryological parameters for prediction of clinical pregnancy.

The overall regression model showed moderate discriminative ability (AUC ≈ 0.70), demonstrating that fertilization- and embryo-related factors independently contribute to predicting clinical pregnancy in IVF/ICSI cycles (Figure 3).

The negative association between excessive oocyte yield and pregnancy supports the concept that hyper-response to ovarian stimulation may compromise oocyte quality or embryo–endometrial synchrony. To identify independent determinants of pregnancy, a multivariate logistic regression model was constructed including the number of 2PN embryos, total embryos, and total oocytes as covariates (Table 5).

**Table 5:** Multivariate logistic regression analysis of predictors for clinical pregnancy.

Predictor	β (Standard Error)	*p*-value	Odds ratio (95% CI)
2PN embryos	0.145 (0.061)	0.0185	1.156 (1.025–1.304)
Total embryos	0.173 (0.066)	0.0099	1.188 (1.044–1.353)
Total oocytes	−0.070 (0.032)	0.0279	0.932 (0.876–0.991)
Constant	−1.624 (0.471)	0.001	—

*Multivariate logistic regression model including total oocyte number, 2PN embryos, and total embryos as covariates. Nagelkerke *R*^2^ = 0.214; overall model *p* < 0.001.

## Discussion

In this large retrospective cohort of 430 women with unexplained infertility undergoing IVF/ICSI, we observed that systemic inflammatory indices derived from complete blood count (CBC)—including NLR, PLR, MLR, SII, SIRI, and PIV—had no predictive value for clinical pregnancy outcomes. Instead, embryologic variables such as the number of two-pronuclei (2PN) embryos and total embryo count emerged as significant and independent predictors of pregnancy success, whereas a higher oocyte yield was inversely related to pregnancy likelihood. When compared with prior literature, our findings diverge from several studies that proposed a prognostic role for hematologic markers in reproductive outcomes.

Several biological pathways may explain why systemic inflammatory indices could theoretically influence IVF outcomes. Pro-inflammatory cytokines can interfere with folliculogenesis, impair granulosa cell function, reduce oocyte competence, and alter endometrial receptivity through effects on immune-cell trafficking, adhesion molecules, and angiogenesis. Increased neutrophil and monocyte activity may also contribute to oxidative stress within the follicular environment, potentially compromising oocyte quality. Conversely, in women with unexplained infertility—who typically lack overt inflammatory disease—systemic cytokine fluctuations may be too subtle to be reflected in peripheral CBC-derived indices, which may explain the absence of an association in our study.

For example, one study examined 132 women with diverse infertility etiologies and found that lower platelet-to-lymphocyte ratios (PLR) were associated with positive β-hCG results (*p* = 0.02); however, this relationship lost significance in multivariate analysis after adjusting for age and MII oocyte count (13). Similarly, Hantoushzadeh et al. reported that lower NLR values were independently associated with higher rates of chemical, clinical, and viable pregnancies (AUC ≈ 0.70), supporting the concept that a reduced systemic inflammatory state may facilitate implantation and early embryonic development (16). However, subsequent investigations and our present results challenge this hypothesis—particularly in more homogeneous, non-inflammatory populations. A study on non-obese women with unexplained infertility (*n* = 246) revealed no significant differences in CBC-derived indices, including NLR, PLR, and MPV, between infertile and control groups, nor any predictive value for implantation or live birth (12).

Likewise, in a cohort of 174 patients categorized by ovarian reserve, only age and FSH predicted diminished ovarian reserve, while NLR and PLR were non-significant (17). These findings collectively imply that inflammation-based hematologic indices may hold prognostic value primarily in heterogeneous or metabolically inflamed populations, but not in normoweight, otherwise healthy women with unexplained infertility. Conversely, studies including women with PCOS or obesity have reported positive associations between CBC-derived indices and IVF outcomes, suggesting that these markers gain predictive strength in pro-inflammatory phenotypes (18). Such differences across populations support the idea that the diagnostic yield of systemic inflammation markers depends largely on the underlying metabolic or immunologic context, rather than representing a universal biological mechanism.

Our study differs from previous reports in several methodological aspects: (i) exclusive inclusion of unexplained infertility, excluding inflammatory conditions such as PCOS or endometriosis; (ii) standardized blood sampling on the trigger day rather than the early follicular phase; and (iii) evaluation of embryological variables in the same model. These differences may explain discrepancies with earlier studies.

Multiple methodological and biological factors may account for inconsistencies across studies.

Population heterogeneity is a key issue—studies identifying positive associations often included women with endometriosis, PCOS, or metabolic syndrome, all of which involve chronic low-grade inflammation. In contrast, our study excluded such conditions, producing a uniform population and thereby minimizing inflammatory variation.

Timing of blood sampling is another possible determinant: earlier studies typically collected samples at baseline or during the early follicular phase, whereas we standardized measurements to the trigger day. As immune cell activity fluctuates throughout ovarian stimulation, single-point measurements might fail to reflect transient peri-implantation immune dynamics. Differences in outcome definitions (chemical, clinical, or viable pregnancy) and the high degree of collinearity among indices (e.g., NLR, PLR, and SII sharing common components) may have further diluted statistical power. Unlike systemic inflammatory indices, our results highlight the robust predictive role of embryologic variables.

Higher 2PN and total embryo counts likely indicate superior oocyte competence, optimal fertilization, and enhanced embryo viability. Interestingly, the negative correlation between excessive oocyte yield and pregnancy parallels previous findings by Sunkara et al., suggesting that overly intense ovarian stimulation may impair developmental potential or disrupt embryo–endometrium synchrony (19). These results emphasize that, in the absence of overt inflammation, reproductive success in IVF is governed primarily by oocyte and embryo quality rather than peripheral immune status. By incorporating composite indices such as SII, SIRI, and PIV, our study extends previous research by exploring more comprehensive representations of immune activation. Nevertheless, none of these indices demonstrated predictive ability, underscoring the limitations of static, peripheral hematologic markers as surrogates for localized reproductive immunology. Future prospective studies incorporating serial immune profiling, cytokine quantification, and endometrial receptivity testing may provide deeper insights into the immune-endocrine interactions underlying implantation success. Additionally, stratification by inflammatory phenotypes such as PCOS or endometriosis could help clarify whether systemic inflammation influences IVF outcomes indirectly through metabolic pathways (18). When interpreted alongside earlier evidence, a consistent pattern emerges: In heterogeneous or metabolically inflamed populations, mild systemic inflammation (elevated NLR/PLR) may adversely affect implantation and pregnancy outcomes. In normoweight and unexplained infertility cohorts, systemic inflammation appears negligible, and hematologic indices lose their predictive value (12). Across all populations, embryo-based variables remain the most reliable determinants of IVF success. Taken together, these findings suggest that while CBC-derived inflammatory indices are of biological interest, their clinical utility in routine IVF prognostication for unexplained infertility remains limited. The major strengths of this study include its large sample size (*n* = 430), the restriction to a homogeneous unexplained infertility cohort, and the standardization of CBC sampling to the ovulation trigger day, minimizing biological and temporal variability.

Unlike previous studies conducted in heterogeneous or metabolically inflamed populations (e.g., PCOS, obesity, or endometriosis), this design allowed a focused evaluation of systemic inflammatory indices independent of confounding inflammatory states (12).

Furthermore, the simultaneous assessment of hematologic, hormonal, and embryologic parameters provides an integrated and multidimensional understanding rarely explored in prior reports (5). The retrospective and single-center design may introduce selection bias and limit external generalizability. Additionally, systemic inflammation was assessed solely through CBC-derived indices without concurrent cytokine or endometrial immune profiling, which might underestimate localized reproductive immunologic activity. Finally, embryo aneuploidy or genetic quality could not be uniformly verified, so embryo count and morphology serve only as indirect surrogates of competence. Taken together, our findings indicate that peripheral hematological inflammatory indices provide little insight into reproductive potential in women with unexplained infertility. Instead, reproductive success is driven mainly by intrinsic factors such as oocyte competence, fertilization efficiency, and embryo developmental capacity. This pattern suggests that embryo-based assessments remain substantially more informative and clinically meaningful than systemic inflammatory markers when predicting IVF outcomes in this population. Future prospective, multi-center studies incorporating longitudinal immune, hormonal, and embryo-based assessments are warranted to validate these findings. In conclusion, systemic inflammatory indices derived from routine CBC parameters—including NLR, PLR, MLR, SII, SIRI, and PIV—did not predict IVF/ICSI outcomes in women with unexplained infertility. These findings align with prior research in non-obese unexplained infertility cohorts showing minimal systemic inflammatory variation and limited predictive power of hematologic markers (1, 5). Conversely, studies conducted in metabolically inflamed populations such as PCOS and obesity have demonstrated associations between these indices and reproductive outcomes (4), highlighting that their predictive value depends on the underlying inflammatory milieu. Our results emphasize that, in otherwise healthy unexplained infertility, embryologic parameters—particularly 2PN and total embryo counts—serve as the most reliable predictors of clinical pregnancy, while excessive oocyte retrieval may be counterproductive. Future prospective studies integrating systemic and localized immune profiling with embryo morphokinetics are essential to clarify the interface between inflammation and reproductive success. Therefore, embryo-based parameters—particularly 2PN and total embryo counts—should be prioritized over hematological inflammatory indices when estimating IVF prognosis in women with unexplained infertility.

## Data Availability

The raw data supporting the conclusions of this article will be made available by the authors, without undue reservation.

## Footnotes

^1^Firdous P, Nissar K, Ali S. Immunogenetic Causes of Infertility. A Molecular Approach to Immunogenetics. Academic Press (2022). p. 227–53. Available online at: https://doi.org/10.1016/B978-0-323-90053-9.00013-0
